# Stable isotope evidence for dietary diversification in the pre-Columbian Amazon

**DOI:** 10.1038/s41598-020-73540-z

**Published:** 2020-10-06

**Authors:** Andre Carlo Colonese, Rachel Winter, Rafael Brandi, Thiago Fossile, Ricardo Fernandes, Silvia Soncin, Krista McGrath, Matthew Von Tersch, Arkley Marques Bandeira

**Affiliations:** 1 BioArCh, Department of Archaeology, University of York, York, YO10 5DD UK; 2 Department of Prehistory, Institute of Environmental Science and Technology (ICTA), Universitat Autònoma de Barcelona, 08193 Bellaterra, Spain; 3 Groningen Institute of Archaeology, University of Groningen, Poststraat 6, 9712 ER Groningen, The Netherlands; 4 Instituto Ambiente Humano (IAH), Av. Germano Moreira, 457, Castelo, Batatais, CP 520, São Paulo, CEP 14300-218 Brazil; 5 Department of Archaeology, Max Planck Institute for the Science of Human History, 07745 Jena, Germany; 6 School of Archaeology, University of Oxford, 1 South Parks Road, Oxford, OX1 3TG UK; 7 Faculty of Arts, Masaryk University, Arne Nováka 1, 60200 Brno-střed, Czech Republic; 8 Programa de Pós-graduação Em Cultura E Sociedade, Programa de Pós-graduação Em Desenvolvimento E Meio Ambiente de Ecossistemas Costeiros e, Departamento de Oceanografia E Limnologia, Universidade Federal Do Maranhão, Av. dos Portugueses, 1966 Bacanga, São Luís, CEP 65080-805 Brazil

**Keywords:** Neuroscience, Biomarkers, Medical research, Neurology, Nanoscience and technology

## Abstract

Archaeological research is radically transforming the view that the Amazon basin and surrounding areas witnessed limited societal development before European contact. Nevertheless, uncertainty remains on the nature of the subsistence systems and the role that aquatic resources, terrestrial mammalian game, and plants had in supporting population growth, geographic dispersal, cultural adaptations and political complexity during the later stages of the pre-Columbian era. This is exacerbated by the general paucity of archaeological human remains enabling individual dietary reconstructions. Here we use stable carbon and nitrogen isotope analysis of bone collagen to reconstruct the diets of human individuals from São Luís Island (Brazilian Amazon coast) dated between *ca*. 1800 and 1000 cal BP and associated with distinct ceramic traditions. We expanded our analysis to include previously published data from Maracá and Marajó Island, in the eastern Amazon. Quantitative estimates of the caloric contributions from food groups and their relative nutrients using a Bayesian Mixing Model revealed distinct subsistence strategies, consisting predominantly of plants and terrestrial mammals and variably complemented with aquatic resources. This study offers novel quantitative information on the extent distinct food categories of polyculture agroforestry systems fulfilled the caloric and protein requirements of Late Holocene pre-Columbian populations in the Amazon basin.

## Introduction

Archaeological investigations in lowland South America are radically transforming our understanding of long-term socio-ecological systems in the Neotropics and dismissing the view that the Amazon basin witnessed limited socio-economic development in pre-Columbian times^1^. Recent studies reveal a complex picture during the Late Holocene, marked by increased archaeological visibility associated with groups producing distinct material cultures and engaging in diversified subsistence strategies^1,2^ with extensive transformation of soils^3^, vegetation composition^4^, and landscapes^5–7^.

During the Late Holocene specialized land-use systems based on agriculture or the management of aquatic resources coexisted with more versatile subsistence strategies founded on polyculture agroforestry^8,9^, with substantial temporal and spatial variability across the basin^2^. Evidence from coastal areas is relatively limited, but equally diversified. Plant cultivation with extensive raised fields has been documented in seasonally flooded savannahs of the northern Amazon (The Guianas)^10^, while fishing-based economies supported by water management systems, complemented with plant gathering and possibly some cultivation, occurred in some areas of the eastern Amazon (Marajó Island)^11,12^. Numerous shell middens along the coast suggest that food security was also achieved through the exploitation of estuarine and marine resources by groups producing some of the earliest ceramic artefacts in Amazonia^13^.

Despite the growing interest in the origin and changing nature of pre-Columbian economies^8^, attempts to comprehend their impacts on individual diets have been comparatively limited^12,14–17^. Moreover, while the role of agriculture in supporting population growth and the establishment of sedentary settlements in the recent pre-Columbian era has been the subject of contentious debate^2,11^, the importance of aquatic resources has received comparatively little attention^18^. Aquatic resources are often invoked as economically important food items to both coastal and riverine populations^7,18–22^, but their relative contribution to diet is poorly understood and surrounded by controversial generalizations^23–26^. Uncertainties also remain regarding the extent to which plants (cultivated, gathered) and terrestrial animals fulfilled the energy requirements of specialized and mixed subsistence systems. Their potential intensification and role in sustaining dense populations during the later stages of the pre-Columbian era is still a matter of debate^2^. Stable carbon and nitrogen isotope analysis of bone collagen is a well established approach for deriving dietary information from human, faunal, and plant remains^27–29^ and has been employed to reconstruct pre-Columbian subsistence economies across much of South America^30–32^. However, due to the paucity of archaeological human remains there have been limited applications on past populations in the Amazon basin until now^12,14–17^, with most studies lacking direct and robust chronological information, as well as contextual isotopic baselines^12,15^.

**Figure 1:**
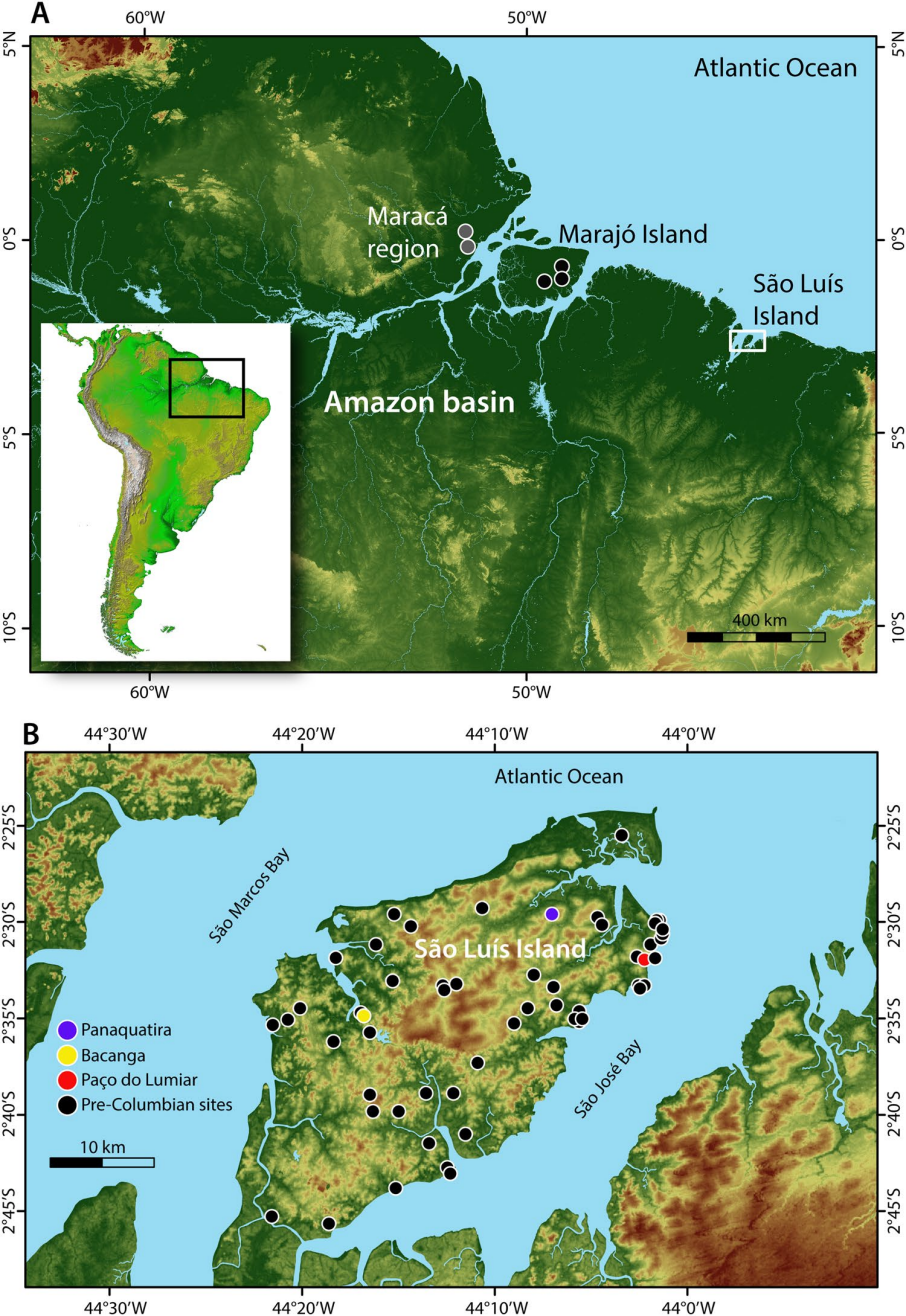
The study area in the eastern Amazon. (**A**) The location of São Luís Island and the approximate location of Late Holocene sites at Maracá region (Gruta das Caretas and Gruta do Pocinho) and on Marajó Island (Teso dos Bichos, Monte Carmelo and Matinadas). (**B**) Detailed location of pre-Columbian sites on São Luís Island, including the sites of Panaquatira, Bacanga and Paço do Lumiar. Maps generated using ArcGIS 10.7 (https://desktop.arcgis.com/en/) and Adobe Illustrator CS6 (https://www.adobe.com/es/products/illustrator.html), on data publicly available from NASA/JPL/NIMA (South American), the Brazilian Institute of Geography and Statistics (IBGE), the National Institute for Space Research (DPI/INPE), and the Brazilian Agricultural Research Corporation (Embrapa) (detailed maps of Amazonia and São Luís Island).

Here we offer a novel contribution to unravel pre-Columbian diets in lowland Amazonia and specifically the role of fish as a source of dietary protein. We analyzed the stable carbon and nitrogen isotope composition of bone collagen from five human individuals from archaeological sites (Panaquatira, Bacanga, and Paço do Lumiar) on São Luís Island, located off the Brazilian Amazon coast (Fig. 1). Four individuals were directly radiocarbon dated (AMS) to between *ca.* 1800 to 1000 cal BP and all were recovered from archaeological contexts associated with distinct ceramic traditions, along with terrestrial mammal, fish, and shellfish remains. The proportional contributions of fish, terrestrial mammals and plants to diet were estimated using Bayesian Stable Isotope Mixing Models (BSIMMs), which provide quantitative estimates of relative caloric and protein contributions from available food groups. Pre-Columbian human remains are rare in the Amazon basin and the obtained results offer the most robust and chronologically secure information on individual diets in pre-Columbian lowland Amazonia to date.

## Results

### Stable isotope analysis and Bayesian dietary reconstruction

Six human individuals were recovered from the archaeological shell middens of Bacanga (n = 1), Paço do Lumiar (n = 1) and Panaquatira (n = 4). The individuals were fragmented and mostly disarticulated, preventing accurate sex and age identification. Four individuals were directly radiocarbon dated using AMS, with the calibrated ages corrected for the average relative contribution of marine carbon to collagen estimated by the BSIMMs (Table 1; see Methods for details).

**Table 1:** Radiocarbon dates of human individuals from Bacanga (BCG), Paço do Lumiar (PDL) and Panaquatira (PNQ). The radiocarbon dates were calibrated (BP) using a combination of the marine (Marine13) and terrestrial (SHCal13) curves in OxCal v4.3, taking into account the average relative contribution of marine carbon to collagen estimated by the BSIMMs.

Site	Individual	Lab code	^14^C yr BP	Material	Cal yr BP (2σ)	Marine C in collagen (%)
BCG	BC1	Beta-341017	1910 ± 30	Collagen	1840–1700	12.96
PDL	PDL2	Beta-341018	1290 ± 30	Collagen	1190–990	15.43
PNQ	PNQ1A	Beta-341020	1910 ± 30	Collagen	1830–1620	17.03
PNQ	PNQ3	Beta-341019	1800 ± 30	Collagen	1710–1540	17.03

At the shell midden of Bacanga one infant individual (BC1) was found in direct association with a ceramic vessel from the Mina tradition, faunal remains, and blocks of ferruginous rocks, and was directly dated to 1840–1700 cal BP (2σ). At Paço do Lumiar, one adult individual (PDL2), dated to 1190–990 cal BP (2σ), was found at the base of the shell midden, with ceramic fragments stylistically comparable to Incised Rim and Mina traditions. At Panaquatira (PNQ), a double burial with one adult (PNQ1A) and one infant (PNQ1B) was found within a ceramic funerary urn with Polychrome decoration, surrounded by stones and shells, emerging from a superficial dark soil overlying the shell midden. The adult (PNQ1A) was dated to 1830–1620 cal BP (2σ). Another adult individual (PNQ2) was found disarticulated and buried with shells and stone tools in a funerary urn with Polychrome decoration, in a dark soil covering the shell midden. This individual did not produce collagen for radiocarbon dating or stable isotope analysis. Another adult individual (PNQ3) was found disarticulated at the base of the shell midden in association with ceramic fragments of Mina tradition and shell beads, and was dated to 1710–1540 cal BP (2σ).

**Figure 2:**
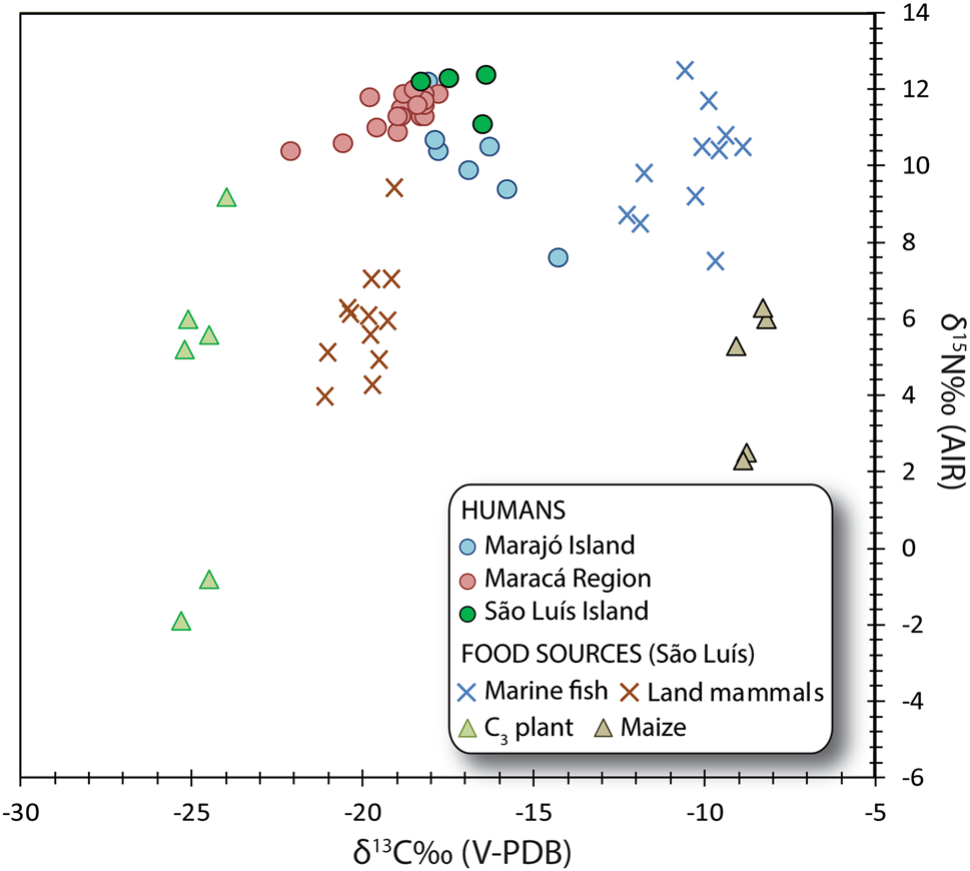
*δ*^13^C and *δ*^15^N values of humans and faunal remains from São Luís Island (Panaquatira, Paço do Lumiar, Bacanga) and from other sites in the Amazon River delta (Maracá region and Marajó Island). Modern plants from São Luís Island (C_3_ crops and maize) are also reported.

Faunal material was collected from the site of Panaquatira (PNQ). The δ^13^C and δ^15^N values of terrestrial mammals (n = 12) ranged from − 21.1 to − 19.1 ‰ and from + 4.0 to + 9.4 ‰, respectively (Fig. 2). The analyzed specimens that could be taxonomically identified (paca, cavy, agouti, brocket deer) were predominantly herbivorous and all *δ*^13^C values fell within the range associated with environments dominated by C_3_ plants^33^, with some amount of water stress that could be expected in the transitional zone between humid and savannah forest^34^. The *δ*^13^C and *δ*^15^N values for fish (n = 11) were statistically distinguishable from terrestrial mammals (n = 23, *p* < 0.01, F = 617.8 for *δ*^13^C, *p* < 0.01, F = 44.52 for *δ*^15^N, One-way Anova) and ranged from − 12.3 to − 8.9 ‰ and from + 7.5 to + 12.5 ‰, respectively. Some of the remains could be identified as catfish (Ariidae), which is a common taxa in the region^35^. The high *δ*^13^C values observed are typical of fish from marine ecosystems^36^ and are also comparable with other marine taxa from the southwestern Atlantic Ocean^30,37^. Our marine samples show *δ*^13^C values considerably higher than modern fish from mangrove environments along the Amazon coast^38^, suggesting a larger contribution of marine phytoplankton to PNQ’s marine food web. We exclude that fish *δ*^13^C values at PNQ could reflect the input of C_4_ aquatic macrophytes since their contribution to fish diet is negligible in this region^39^. Fish *δ*^15^N values reflect diets based on a mixture of benthic organisms (crustaceans, polychaetes) and detritus^38^.

Modern plant *δ*^13^C and *δ*^15^N values from São Luís Island varied considerably within and between taxa (Supplementary information 1). The *δ*^13^C values of bulk plant (tuber and kernel) were corrected for the Suess effect (+ 2.13 ‰) (see Methods for details). Maize (*Zea mays*) had *δ*^13^C and *δ*^15^N values ranging from − 9.1 to − 8.2 ‰ and from + 2.3 to + 6.3 ‰, respectively. Manioc and macaxeira (both *Manihot esculenta*) and sweet potato (*Ipomoea batatas*) had comparable *δ*^13^C values, from − 25.3 to − 24.0 ‰, but contrasting *δ*^15^N values. A single sweet potato had a *δ*^15^N value of + 9.2 ‰, while manioc tubers displayed a larger variability, ranging from − 1.9 to + 6.0 ‰. The reason for the differences in *δ*^15^N values among manioc and macaxeira samples is unclear but may arise from the differential use of fertilizers and/or cultivation of macaxeira in soils with high ^14^N volatilization or mineralization in the context of swidden agriculture^40^.

The *δ*^13^C values for human individuals from São Luís Island ranged from − 16.4 ‰ (PNQ1A and PNQ3) to − 18.3 ‰ (BC1) (Fig. 2). The *δ*^15^N values ranged from + 11.1 ‰ (PNQ1B) to + 12.4 ‰ (PNQ3). There were no substantial differences in the *δ*^15^N values between adults and infants, and the lack of sex identification prevented further consideration of the sex and gender dimensions of diet. Nevertheless, it is worth noting that *δ*^15^N values for the two infants (PNQ1B and BC1) could also reflect some level of breastfeeding effect^41^, implying that some young-adult lactating women had even lower *δ*^15^N values compared to those reported here. Overall the isotope results indicate mixed subsistence strategies with substantial contribution of C_3_ plants, terrestrial mammalian game, marine fish and possibly maize (although a number of C_4_ plants occur in the Amazon^42^).

The stable isotope values for the individuals from São Luís Island show significant differences compared to Late Holocene pre-Columbian groups that inhabited the eastern Amazon basin (Fig. 2). A number of human individuals from the sites of Teso dos Bichos, Monte Carmelo, Matinadas and an unknown site on Marajó Island (n = 7) have collagen *δ*^13^C and *δ*^15^N values consistent with mixed contributions from freshwater fish, C_3_, and C_4_ plant protein^12^. The precise chronology of these samples is unknown^12^, but they may be attributed to stratified chiefdom-level societies (Marajoara Phase) which supposedly intensified the exploitation of freshwater resources at *ca.* 1600–650 BP, a process that supported or catalyzed considerable social complexity around the control of aquatic resources in the region^43^. Freshwater fish was also frequently consumed by Late Holocene individuals (n = 17) from Gruta das Caretas and Gruta do Pocinho in Maracá, on the north bank of the Amazon river, dating to *ca.* 500 BP. The bulk collagen *δ*^13^C and *δ*^15^N values for these individuals were interpreted as resulting from diets based on freshwater resources, together with C_3_ plants, and possibly also some contributions from C_4_ or CAM crops^15^.

In general, the *δ*^13^C values at São Luís Island were significantly higher compared with those from the Maracá region (n = 22, *p* < 0.05, Kruskal–Wallis), but not significantly different from those of Marajó Island (n = 12, *p* > 0.05, F = 0.1734, One-way ANOVA). Differences between São Luís Island and Maracá can be attributed to the consumption of marine resources at São Luís Island. The high *δ*^13^C values observed at Marajó Island, instead, could indicate the contribution of maize to dietary proteins^12^. The *δ*^15^N values at São Luís were significantly higher compared to both Maracá (n = 22, *p* < 0.05, Kruskal–Wallis) and Marajó Island (n = 12, *p* < 0.05, Kruskal–Wallis), suggesting a greater consumption of terrestrial or aquatic animal protein at São Luís and/or regional isotopic differences in resource baselines^44^. These mixed dietary regimes, large variations in the macronutrient compositions of foods, and uncertainties in food isotopic values and in diet-to-consumer isotopic offsets limit the use of simple linear mixing models. The multiple sources of uncertainty can be more efficiently handled by employing Bayesian Stable Isotope Mixing Models (BSIMMs) that express dietary contribution probability distributions^45–47^. To estimate the caloric contributions for the São Luís individuals from four food groups (terrestrial C_3_ plants, maize, terrestrial mammals, and marine fish) and for both Maracá region and Marajó Island (terrestrial C_3_ plants, maize, terrestrial mammals, and freshwater fish/reptile) we employed the Bayesian mixing model FRUITS^45^. There is no evidence that individuals from both Maracá region and Marajó Island exploited marine resources, however freshwater fish remains were reported for the site of Teso dos Bichos (Marajó Island), along with plants and a few terrestrial mammals^12,15^. The model in FRUITS accounts for the uncertainties in consumer and food isotopic values, macronutrient composition, diet-to-consumer isotopic offsets, and dietary routing. The latter accounts for non-protein contributions (carbohydrates and lipids) to consumer *δ*^13^C collagen, necessary for higher-accuracy dietary estimates^48^. Two dietary estimates were generated: the relative caloric contribution from each food group, and the relative caloric contribution from the protein-only component of each food group. These two estimates differ since the macronutrient composition of the food groups varies. Animal food groups typically have a considerably higher protein content when compared to plant foods, thus, caloric contributions may be predominantly from plants while animal sources simultaneously constitute the main source of protein. In the following sections, *caloric estimates/contributions* refers to food caloric contributions and *protein estimates/contributions* refers to the caloric contributions from the protein-only component of the food groups (see Material and Methods for model implementation details).

**Figure 3:**
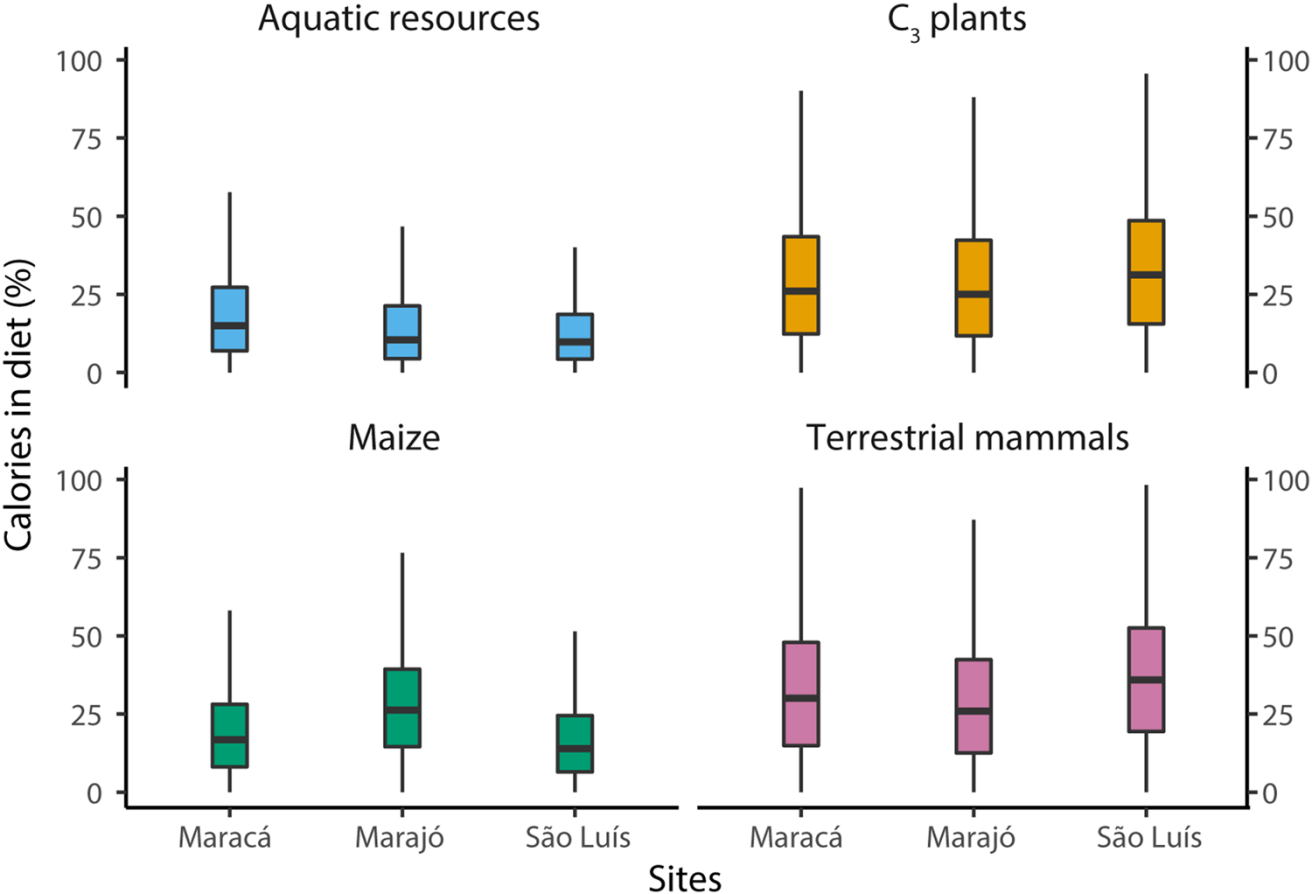
Box and whiskers plot summarizing the probability distribution of the relative caloric contributions from distinct food groups to individual diets at Maracá, Marajó Island and São Luís Island. The horizontal lines correspond to the median and the hinges to the 25th (Q1) and 75th (Q3) percentiles. The whiskers extend from the hinge to the smallest or largest observation greater than or equal to − 1.5 * IQR or less than or equal to + 1.5 * IQR, respectively.

The dietary estimates generated by FRUITS for individuals at São Luís Island indicated that the majority of calories were supplied by terrestrial mammals (median 36%, Q1 19% and Q3 53%) and/or C_3_ plants (median 31%, Q1 16% and Q3 49%), followed by maize (median 14%, Q1 7% and Q3 25%) and marine fish (median 10%, Q1 4% and Q3 19%) (Fig. 3). There was considerable overlap between caloric output from terrestrial mammals and C_3_ plants for both individual and group estimates. This lack of estimate resolution is due to insufficient separation of the distribution of isotopic values characterizing the two food groups and to isotopic equifinality, that is, varying combinations of food contributions that may result in the same consumer isotopic values.

**Figure 4:**
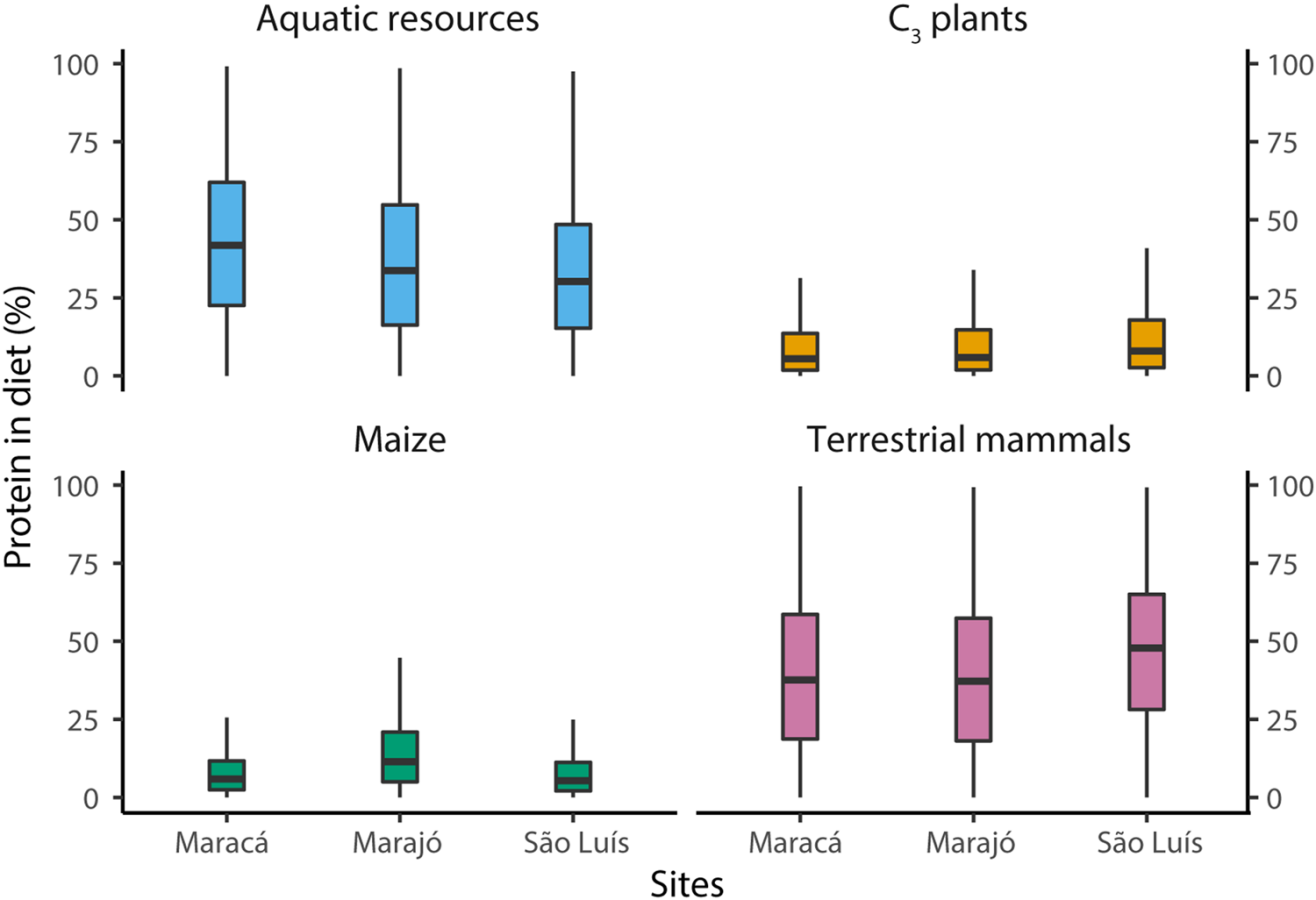
Box and whiskers plot summarizing the probability distribution of the relative caloric contribution from the protein-only component from distinct food groups to individual diets at Maracá region, Marajó Island and São Luís Island. The horizontal lines correspond to the median and the hinges to the 25th (Q1) and 75th (Q3) percentiles. The whiskers extend from the hinge to the smallest or largest observation greater than or equal to -1.5 * IQR or less than or equal to + 1.5 * IQR, respectively.

The model estimated that terrestrial mammals were the main source of dietary protein for all individuals (median 48%, Q1 28% and Q3 65%), followed by marine fish (median 30%, Q1 15% and Q3 48%), C_3_ plants (median 8%, Q1 3% and Q3 18%), and then maize (median 5%, Q1 2% and Q3 11%) (Fig. 4). Compared to relative caloric estimates, protein contributions achieved a higher precision (see also Supplementary information 2 and 3). In summary, individuals at São Luís Island relied primarily on terrestrial resources for both calories and proteins.

For individuals from Marajó Island, FRUITS estimates assigned the majority of dietary calories to terrestrial mammals (median 26%, Q1 13% and Q3 42%), and/or C_3_ plants (median 25%, Q1 12% and Q3 42%) and/or maize (median 26%, Q1 15% and Q3 39%), with considerable overlap among estimates. Freshwater fish/reptiles offered the smallest contribution of all food groups (median 10%, Q1 4% and Q3 21%). It is worth noting that one individual (MrJ3, from Teso dos Bichos) had maize as the prevailing source of calories (average 34 ± 14%). Terrestrial mammals (median 37%, Q1 18% and Q3 57%) and/or freshwater fish/reptiles (median 34%, Q1 16% and Q3 55%) were the main sources of dietary protein, although with estimates overlapping considerably for most of the individuals. These were then followed by maize (median 11%, Q1 5% and Q3 21%) and C_3_ plants (median 6%, Q1 2% and 15%).

Similarly, the probability distributions generated by FRUITS for individuals from the Maracá region revealed that the majority of dietary calories were sourced from terrestrial mammals (median 30%, Q1 15% and Q3 48%) and/or C_3_ plants (median 26%, Q1 12% and Q3 43%), with some overlap in the estimates. These were followed by maize (median 17%, Q1 8% and Q3 28%) and freshwater fish/reptiles (median 15%, Q1 7% and Q3 27%), although for some individuals these two food groups also overlapped considerably. Dietary proteins were obtained from freshwater fish/reptiles (median 42%, Q1 23% and Q3 62%) and/or terrestrial mammals (median 38%, Q1 19% and Q3 59%), followed by contributions from maize (median 6%, Q1 2% and Q3 12%) and C_3_ plants (median 5%, Q1 2% and Q3 14%).

## Discussion

The results from São Luís Island, along with the review of previously published *δ*^13^C and *δ*^15^N values using a BSIMMs approach, reveal distinct adaptive strategies in the eastern Amazon basin during the Late Holocene. The differential dietary contributions from terrestrial mammal and C_3_ plants at São Luís Island could not be efficiently resolved by BSIMMs due to the isotopic similarity of these sources and the isotopic equifinality resulting from the mixed contributions from all food groups. However, model estimates adequately distinguished the relative contribution of marine fish from the terrestrial food groups (terrestrial mammals, C_3_, and maize). Despite the proximity to marine resources and contextual zooarchaeological evidence for fishing, model estimates reveal that terrestrial mammals provided greater amounts of dietary proteins to individuals at São Luís Island compared to marine fish. These results call into question the widespread assumption that aquatic resources (e.g.fish, reptiles) were the main economic component, or source of protein, to pre-Columbian populations living in proximity to productive aquatic environments in lowland Amazonia^20–24^. However, it is necessary to acknowledge that only a small number of samples were analysed.

Freshwater fish/reptiles and/or terrestrial mammals provided the majority of dietary protein to populations located at the mouth of the Amazon river (Marajó Island and Maracá), but uncertainties remain on the relative contributions from these two food groups. According to Roosevelt^12^, freshwater fish dominated faunal remains at the site of Teso dos Bichos on Marajó Island, while remains of terrestrial mammalian game were rare. However, for most of the sites on Marajó Island and in the Maracá region there is no available information on faunal remains^15^. This lack of contextual faunal and botanical data, along with uncertainties associated with the local isotope baselines, limit the possibility of obtaining precise dietary estimates. Model estimates, however, do indicate that the majority of caloric contributions were from terrestrial mammals and plants, and not from freshwater resources. While fish may have been a dominant source of protein, our results suggest that considerable investments were allocated to hunting, forest management, and plant cultivation, as shown by other lines of evidence across the Amazon basin^1,4,10,49^.

Maize, a widespread C_4_ crop in the Amazon by the Late Holocene^50^, was not a staple for the sampled individuals, but was likely consumed within the broad spectrum of resources exploited by distinct agroforestry systems. The relatively high consumption of C_4_ plants (possibly maize) by some individuals at Marajó Island is significant though, and may reopen a contentious debate on the role that plant cultivation had in supporting the emergence of political complexity during the Marajoara Phase^11,12^. While we recognize that dietary estimates can be further refined with increasing control of contextual faunal and plant baselines^51^ and isotopic analyses of single amino acids^29^, the results presented herein constitute the most robust source of information on individual diets in the pre-Columbian Amazon and corroborate the growing consensus that diversified subsistence economies fuelled cultural, demographic and environmental transformations in the eastern Amazon basin during the Late Holocene.

## Methods

### Geographic and archaeological contexts

São Luís Island, in the state of Maranhão, is approximately 832 km^2^ and is part of the Maranhense Gulf geological-geomorphological feature which forms a large and complex estuarine system delimited by the bays of São Marcos and São José (Fig. 1). Lying in the transition between Amazon and Northeastern vegetation^52^, the region has a tropical climate with dry (January to June) and rainy (June to December) seasons, with species from the Amazon forest, caatinga, and cerrado biomes, and extensive restinga and mangrove ecosystems along the coast which are important nurseries for aquatic organisms^53^. The coastal environment is highly dynamic and shows relatively high biological productivity and biomass with limited seasonal variability due to the combination of semidiurnal macro-tidal regimes, mangrove vegetation, and freshwater input (notably in São Marcos Bay)^35^. This has favoured fish and shellfish exploitation by groups on the island since at least the Middle and Late Holocene^54^, during the spread of ceramic artefacts associated with the Mina tradition. The archaeological shell midden of Bacanga contains faunal evidence of fishing in marine and estuarine environments at that time, including taxa of different trophic positions (e.g. sharks, catfish). Marine resources were also exploited by groups using ceramic vessels similar to the Amazonian Incised Rim and possibly the Polychrome tradition during the Late Holocene, the latter associated with dark soils visibly similar to Amazonian Dark Earths^55^. Evidence of fishing and shellfish collection at such sites are better represented at the shell middens of Paço do Lumiar and Panaquatira^55^. Fishing is well documented historically among local Tupinambá groups in the seventeenth century^56–58^ and currently has major socio-economic and cultural importance on the Island^59^.

### Sample preparation for stable isotopic analysis

Archaeological human and faunal samples from São Luís Island were provided by *Brandi e Bandeira Consultoria Cultural* and the permits for stable isotope analyses were obtained from the *Instituto do Patrimônio Histórico e Artístico Nacional* (IPHAN, protocol no. 01506.00407/2012-14). Rib bones were used for collagen extraction from all six human individuals. The regional faunal isotopic baseline was established with bone collagen of fish (n = 11) and terrestrial mammal (n = 12) remains selected from the dark soil overlying the shell midden of PNQ. Faunal samples were from a range of different skeletal elements and only a few terrestrial species could be taxonomically identified, including agouti (*Dasyprocta punctata*), brocket deer (*Mazama gouazoubira*), lowland paca (*Cuniculus paca*), and cavy (*Kerodon rupestris*). These are predominantly herbivorous animals, feeding on leaves, fruits, tubers, flowers, and bark. Only a few fish remains could be identified as catfish (Ariidae) (Supplementary information 1).

Modern plants (n = 11) were sampled and analyzed for their stable carbon and nitrogen isotope compositions to complement the faunal isotopic baseline. Major neotropical crops including manioc (*Manihot esculenta*, with the local variants *macaxeira* and *mandioca*) and sweet potato (*Ipomoea batatas*) (all C_3_ plants), and maize (*Zea maize*, a C_4_ plant) were acquired from a local small scale market by A. Bandeira at São Luís and Baixada Maranhense in 2018. Before sending the samples for isotope analysis at the University of York, the samples were registered in the *Sistema Nacional de Gestão do Patrimônio Genético e do Conhecimento Tradicional Associado* (SisGen, protocol no. A7BC812) according to article 22 of Decreto nº 8.772, of 11 May 2016. These crops are known to have been cultivated by pre-Columbian groups in lowland Amazonia^60^ and may have constituted a substantial source of dietary energy to populations on São Luís Island (Supplementary information 1).

Collagen was extracted at the BioArCh facilities of the University of York (UK) following the procedure reported in^61^ using a modified Longin method^62^. Bones were cleaned manually and sherds (200–600 mg) demineralized in a 0.6 M HCl solution at 4ºC for 12—72 h. Samples were then rinsed with deionized water (milli-Q®) and immersed in 0.001 M HCl at 80 °C for 48 h. For most samples the supernatant containing the collagen was filtered using Polyethylene Ezee filters (Elkay Laboratories Ltd., 9 mL, pore size 60–90 μm). The samples were then filtered using 30 kDa Amicon® Ultra-4 centrifugal filter units (Millipore, MA, USA), frozen for 24–48 h at − 20ºC, lyophilized, and weighed into tin capsules (1 mg) for stable isotopic analysis.

Plant samples, including tubers of manioc and macaxeira (n = 5) and sweet potato (n = 1), and maize kernels (n = 5), were rinsed in deionized water (milli-Q®), dried and frozen for several hours at − 20 ºC. Samples were lyophilized, homogenized using an agate pestle and mortar, and weighed into tin capsules (2 mg) for bulk stable carbon and nitrogen isotopic analysis.

The collagen samples of four human individuals (PNQ1A, PNQ1B, PDL2, BC1) were analyzed in duplicate by EA-IRMS on a Thermo Finnigan Delta Plus XL in the Department of Archaeological Sciences of the University of Bradford (UK). The collagen sample of PNQ3 and the faunal remains, along with the bulk plant samples, were analyzed in duplicate on a Sercon GSL elemental analyser coupled to a 20–22 mass spectrometer (Sercon, Crewe, UK) at the University of York (UK). At both York and Bradford, accuracy was determined by measurements of international standard reference materials within each analytical run. The obtained values were corrected from the isotopic ratio of the international standards, Vienna Pee Dee Belemnite (VPDB) for carbon and air (AIR) for nitrogen, according to the following equation: *δ*^13^C, *δ*^15^N = (R_sample_/R_standard_)-1^63^, where R = ^13^C/^12^C and ^15^N/^14^ N. At York the overall uncertainties on the measurements of each sample were calculated based on the method of Kragten^64^ by combining uncertainties in the values of the international reference materials and those determined from repeated measurements of samples and reference materials (these are expressed as one standard deviation). Caffeine (IAEA-600), ammonium sulphate (IAEA-N-2), and cane sugar (IA-Cane) international standards were used as reference material in each analytical run. In addition, a homogenised bovine bone was extracted and analysed within the same batch. The in-house collagen standard (bovine control) was also exchanged between laboratories (University of York and University of Bradford) to ensure accuracy.

Collagen was successfully extracted from five human individuals and 23 faunal samples (Table 2 and Supplementary information 1). The extracted collagen had atomic characteristics (C:N ratios, wt%C and wt%N) typical of well-preserved collagen^65–67^. One fish sample had wt%N and wt%C values outside of the acceptable range proposed by Szpak^68^ but had an acceptable C:N ratio and isotope values comparable to the other fish remains, thus it was considered reliable.

**Table 2:** Stable isotope values of bulk collagen of human individuals from Bacanga (BCG), Paço do Lumiar (PDL) and Panaquatira (PNQ). Their ceramic traditions are reported for cultural attribution.

Site	Individual	Age	Sex	Sample	Ceramic tradition	δ^13^C‰	δ^15^N‰	wt%C	wt%N	C:N	Coll yield (wt%)
BCG	BC1	Infant	Undetermined	Rib	Mina	− 18.3	12.2	28.9	10.1	3.3	3.3
PDL	PDL2	Adult	Undetermined	Rib	Mina, Incised Rim	− 17.5	12.3	33.7	9.5	3.2	3.2
PNQ	PNQ1A	Adult	Undetermined	Rib	Painted (Polychrome?)	− 16.5	11.1	40.0	13.8	3.4	2.3
PNQ	PNQ1B	Infant	Undetermined	Rib	Painted (Polychrome?)	− 16.4	12.4	41.3	14.7	3.3	2.0
PNQ	PNQ3	Adult	Undetermined	Rib	Mina	− 16.4	12.4	33.6	11.9	3.2	7.4

### Statistical analysis and Bayesian stable isotope mixing models

Comparisons of *δ*^13^C and *δ*^15^N values between samples were performed using one-way ANOVA or Kruskal–Wallis tests (α = 0.05), after checking for normal distribution with Shapiro–Wilk test for normality (α = 0.05); both tests were performed in PAST 3.x^69^. The relative contributions of different food groups to human diet were estimated using Bayesian Stable Isotope Mixing Models (BSIMMs) in FRUITS 3.1^45^ based on dietary proxies (bone collagen *δ*^13^C and *δ*^15^N values) and food groups (terrestrial mammals, fish and plants) to account for multiple dietary sources, different macronutrient fractions, metabolic routing and uncertainties for all of these parameters. For each food group the average *δ*^13^C and *δ*^15^N values of the nutrient fraction (protein, carbohydrates, lipids) were estimated using offsets between edible fractions and food remains reported in the literature^48,70^. In the case of individuals from São Luís Island, the *δ*^13^C and *δ*^15^N values of the dietary macronutrients (protein and energy) were estimated from the average *δ*^13^C and *δ*^15^N values of terrestrial mammals (n = 12) and fish (n = 11) found at PNQ, using the following offsets—terrestrial mammals: − 2 ‰ (∆^13^C_protein-collagen_), − 8 ‰ (∆^13^C_lipids-collagen_) and + 2 ‰ (∆^15^N_protein-collagen_); fish: − 1 ‰ (∆^13^C_protein-collagen_), − 7 ‰ (∆^13^C_lipids-collagen_) and + 2 ‰ (∆^15^N_protein-collagen_). For plants, bulk *δ*^13^C values were first corrected for the Suess effect. A linear regression using *δ*^13^C values of atmospheric CO_2_ between 1962 and 2008^71^ was used to estimate the atmospheric *δ*^13^C value for 2018 (− 8.6 ‰), when the plants were collected. The difference between 2018 and the value for 1861 (− 6.48 ‰) was then used to correct the bulk plant *δ*^13^C values (+ 2.13 ‰). Plant *δ*^13^C values were then corrected for the offsets of − 2 ‰ (∆^13^C_bulk-protein_) and + 0.5 ‰ (∆^13^C_bulk-carbohydrate_), while assuming that the *δ*^15^N value of plant protein was the same as the average bulk plant *δ*^15^N value. A conservative uncertainty of 1 ‰ was used for most of the *δ*^13^C offsets, except for the average *δ*^13^C values of fish protein and energy (1.5 ‰). Large uncertainties were used for protein *δ*^15^N values of terrestrial C_3_ plants (6 ‰), terrestrial mammals (2 ‰), maize (2 ‰) and fish (2 ‰) to take into account the isotope variation observed between some of these samples (Supplementary information 1).

Bayesian estimates were also obtained for previously published collagen *δ*^15^N and *δ*^13^C values of 24 human individuals from the Maracá region (Gruta das Caretas: GdC, and Gruta do Pocinho: GdP)^15^ and Marajó Island (MrJ: unknown site, Teso dos Bichos, Monte Carmelo and Matinadas)^12^, at the mouth of the Amazon River. The model used for these sites differed from São Luís Island in regards to the faunal isotope baseline. Due to the lack of contextual isotopic baselines, their dietary macronutrients were estimated on the *δ*^13^C and *δ*^15^N values of modern terrestrial mammals (n = 8) and freshwater resources (fish and reptiles; n = 27) sampled from Guiana, Lower Orinoco and Upper Amazon^15,72,73^, and from the modern plants collected at São Luís Island (Supplementary information 2). The *δ*^13^C values of modern fauna were first corrected for the Suess effect using the procedure reported above for their year of capture, and then both *δ*^13^C and *δ*^15^N values were corrected for isotopic fractionation between tissue (muscle and bone collagen) and macronutrients using the aforementioned offsets (Supplementary information 1).

A concentration-dependent and routed model was selected for the Bayesian estimations^49^, where lipids and carbohydrates were aggregated as energy in the model input. The nitrogen isotopes were assumed to be sourced only from proteins (100%), but the carbon isotopes could have derived from carbohydrates and lipids with de novo synthesis of non-essential amino acids^28^. We assumed that dietary proteins contributed to 74 ± 4% of bulk collagen carbon, while lipids and carbohydrates provided the remaining 26%^74^. Diet-to-collagen *δ*^13^C offset (+ 4.8 ± 2 ‰) and *δ*^15^N offset (+ 5.5 ± 2 ‰) were taken from Fernandes et al.^74^. Dietary estimations were constrained for a conservative acceptable range for protein intake of > 5% and < 45% of the total calories according to physiological studies^45^. The model inputs and parameters can be found in the Supplementary information 2.

Box and whiskers plots summarizing the posteriors of dietary estimates generated using FRUITS were created using the R statistical software environment (version 4.0.2) and the package ggplot2 (version 3.3.2)^75^, with McGill et al.^76^ variations of Box Plots. For these, the horizontal lines correspond to the median and the hinges to the 25th (Q1) and 75th (Q3) percentiles. The whiskers extend from the hinge to the smallest or largest observation greater than or equal to − 1.5 * IQR (interquartile range) or less than or equal to + 1.5 * IQR, respectively; outliers were excluded. Summary statistics of the dataset were performed using dplyr version 1.0.1 and reported in the Supplementary information 3.

Finally, we assumed that maize, a plant using a C_4_ photosynthetic pathway, was consumed by groups at São Luís Island and at the mouth of the Amazon River during the Late Holocene. This assumption is reasonably supported by the presence of maize in the Amazon basin since the Middle Holocene^50,77^, and by increasing evidence of maize in archaeological and lake sedimentary records in the central and northern Amazon during the Late Holocene^10,78^.

The radiocarbon dates from São Luís Island, obtained directly from human individuals using Accelerator Mass Spectrometry (AMS), were calibrated (BP) using a combination of the marine (Marine13^79^) and terrestrial (SHCal13^80^) curves in OxCal v4.3^81^, taking into account the average relative contribution of marine carbon to collagen estimated by the BSIMMs. We estimated the local marine radiocarbon reservoir correction value (∆R) at São Luís Island using data from the Marine Reservoir Correction Database^82^. The ∆R point data from the Marine Reservoir Correction Database was used to generate a smooth surface distribution of marine ∆R values using the Bayesian model AverageR developed within the Pandora & IsoMemo initiatives (https://isomemoapp.com). AverageR is a generalized additive mixed model that employs a thin plate regression spline. Further details on the model can be found in Cubas et al.^83^. The local ∆R (10 ± 41 ^14^C years) was estimated for a point centred at São Luís Island and a radius of 50 km. Calibrated dates were rounded to 10.

## Supplementary information

Supplementary file1

Supplementary file2

Supplementary file3

## Data Availability

All the data presented and discussed in this paper is provided in the tables and supplementary information.
