# Supplementary material for: Stable isotope evidence for dietary diversification in the pre-Columbian Amazon
Source: Sci Rep. 2020 Oct 6;10:16560. doi: 10.1038/s41598-020-73540-z (PMC7539003; doi:10.1038/s41598-020-73540-z)
Supplement: Supplementary file 1 — Supplementary file1 [file 41598_2020_73540_MOESM1_ESM.docx]

**Stable isotope evidence for dietary diversification in the pre-Columbian Amazon**

Andre Carlo Colonese, Rachel Winter, Rafael Brandi, Thiago Fossile, Ricardo Fernandes, Silvia Soncin, Krista McGrath, Matthew Von Tersch, Arkley Marques Bandeira

Supplementary information 1

| **Site** | **Taxa** | **Common name** | **δ^13^C‰** | **δ^15^N‰** | **wt%C** | **wt%N** | **C/N** | **Col yield (wt%)** |
| --- | --- | --- | --- | --- | --- | --- | --- | --- |
| PNQ | *Cuniculus* sp. | paca | -19.9 | 6.1 | 37.6 | 13.2 | 3.3 | 2.7 |
| PNQ | *Cuniculus* sp. | paca | -19.2 | 7.0 | 34.4 | 12.2 | 3.3 | 2.0 |
| PNQ | *Kerodon* sp. | cavy | -19.1 | 9.4 | 32.3 | 11.7 | 3.2 | 3.1 |
| PNQ | *Kerodon* sp. | cavy | -19.3 | 5.9 | 24.4 | 8.7 | 3.3 | 1.7 |
| PNQ | *Dasyproct* sp. | cutia | -19.5 | 4.9 | 35.6 | 12.6 | 3.3 | 1.5 |
| PNQ | *Dasyproct* sp. | cutia | -19.7 | 4.3 | 42.6 | 15.2 | 3.3 | 3.0 |
| PNQ | *Mazama* sp. | brocket deer | -20.5 | 6.3 | 35.5 | 12.7 | 3.3 | 1.1 |
| PNQ | *Mazama* sp. | brocket deer | -20.4 | 6.1 | 42.5 | 15.4 | 3.2 | 2.4 |
| PNQ | Terrestrial mammal | unknown | -21.1 | 4.0 | 34.7 | 12.4 | 3.3 | 1.4 |
| PNQ | Terrestrial mammal | unknown | -21.1 | 5.1 | 44.1 | 15.5 | 3.3 | 4.7 |
| PNQ | Terrestrial mammal | unknown | -19.8 | 7.0 | 43.8 | 15.6 | 3.3 | 2.4 |
| PNQ | Terrestrial mammal | unknown | -19.8 | 5.6 | 37.4 | 13.5 | 3.2 | 3.3 |
| PNQ | Ariidae | catfish | -8.9 | 10.5 | 42.0 | 15.0 | 3.2 | 1.3 |
| PNQ | Ariidae | catfish | -10.1 | 10.5 | 18.9 | 6.7 | 3.3 | 1.2 |
| PNQ | Ariidae | catfish | -10.3 | 9.2 | 25.6 | 9.2 | 3.3 | 2.4 |
| PNQ | fish | unknown | -9.7 | 7.5 | 15.4 | 5.6 | 3.2 | 2.2 |
| PNQ | fish | unknown | -11.9 | 8.5 | 13.8 | 5.0 | 3.3 | 2.0 |
| PNQ | fish | unknown | -10.6 | 12.5 | 40.3 | 14.2 | 3.3 | 1.9 |
| PNQ | fish | unknown | -11.8 | 9.8 | 12.2 | 4.1 | 3.5 | 1.1 |
| PNQ | fish | unknown | -9.4 | 10.8 | 23.6 | 8.4 | 3.3 | 1.0 |
| PNQ | fish | unknown | -9.6 | 10.4 | 15.2 | 5.2 | 3.4 | 1.2 |
| PNQ | fish | unknown | -12.3 | 8.7 | 21.5 | 7.7 | 3.2 | 1.7 |
| PNQ | fish | unknown | -9.9 | 11.7 | 22.1 | 8.1 | 3.2 | 4.1 |

Table 1: Stable isotope values of bulk collagen of terrestrial and marine faunal remains from Panaquatira (PNQ).

| **Taxa** | **Common name** | **δ^13^C‰** | **δ^15^N‰** | **wt%C** | **wt%N** |
| --- | --- | --- | --- | --- | --- |
| *Manihot esculenta* | Manioc | -25.1 | 6.0 | 40.7 | 0.4 |
| *Manihot esculenta* | Manioc | -25.2 | 5.2 | 40.9 | 0.3 |
| *Manihot esculenta* | Macaxeira | -24.5 | 5.6 | 40.7 | 0.4 |
| *Manihot esculenta* | Macaxeira | -24.5 | -0.8 | 40.8 | 0.2 |
| *Manihot esculenta* | Macaxeira | -25.3 | -1.9 | 40.5 | 0.2 |
| *Ipomoea batatas* | Sweet potato | -24.0 | 9.2 | 41.0 | 0.8 |
| *Zea maize* | Zea maize | -9.1 | 5.3 | 42.1 | 3.0 |
| *Zea maize* | Zea maize | -8.8 | 2.5 | 41.5 | 2.7 |
| *Zea maize* | Zea maize | -8.2 | 6.0 | 42.3 | 3.3 |
| *Zea maize* | Zea maize | -8.9 | 2.3 | 41.7 | 2.8 |
| *Zea maize* | Zea maize | -8.3 | 6.3 | 41.4 | 3.7 |

Table 2: Stable isotope values of bulk plant (tuber and kernel) of specimens acquired in 2018 at São Luís Island and Baixada Maranhense. The δ^13^C values were corrected for the Suess effect (+ 2.13 ‰). See the manuscript for details.

| Food sources | Fraction | δ^13^C‰ | δ^15^N‰ |
| --- | --- | --- | --- |
| C_3_ plant | Protein | -26.8 ± 1.0 | +2.6 ± 6.0 |
| C_3_ plant | Carbohydrates | -24.3 ± 1.0 | - |
| C_4_ plant | Protein | -10.7 ± 1.0 | +4.5 ± 2.0 |
| C_4_ plant | Energy | -8.2 ± 1.0 | - |
| Terrestrial mammals | Protein | -21.9 ± 1.0 | +8.2 ± 2.0 |
| Terrestrial mammals | Energy | -27.9 ± 1.0 | - |
| Marine fish | Protein | -11.4± 1.5 | +12.0 ± 2.0 |
| Marine fish | Energy | -17.4 ± 1.5 | - |

Table 3: Isotope values of food source fractions for individual from São Luís Island derived from measured faunal bulk collagen and plant bulk isotope values using offsets reported in the text. Plant δ^13^C values were corrected for the Suess effect (see manuscript).

| Food sources | Fraction | δ^13^C‰ | δ^15^N‰ |
| --- | --- | --- | --- |
| C_3_ plant | Protein | -26.8 ± 1.0 | +2.6 ± 6 |
| C_3_ plant | Carbohydrates | -24.3 ± 1.0 | - |
| C_4_ plant | Protein | -10.7 ± 1.0 | +4.5 ± 2.0 |
| C_4_ plant | Energy | -8.2 ± 1.0 |  |
| Terrestrial mammals | Protein | -24.0 ± 1.0 | +6.7 ± 1.9 |
| Terrestrial mammals | Energy | -26.0 ± 1.0 | - |
| Freshwater resources | Protein | -25.3 ± 3.0 | +11.0 ± 2.1 |
| Freshwater resources | Energy | -31.4 ± 3.0 | - |

Table 4: Isotope values of food source fractions for individual from Maracá and Marajó Island derived from published faunal bulk collagen and measured plant bulk isotope values using offsets reported in the text. Plant and faunal δ^13^C values were corrected for the Suess effect (see manuscript).
